# Payday lenders and premature mortality

**DOI:** 10.3389/fpubh.2022.993585

**Published:** 2022-10-18

**Authors:** Megan Agnew, Megan Doherty Bea, Terri Friedline

**Affiliations:** ^1^Department of Population Health Sciences, University of Wisconsin-Madison, Madison, WI, United States; ^2^Department of Consumer Science, University of Wisconsin-Madison, Madison, WI, United States; ^3^University of Michigan, Ann Arbor, MI, United States

**Keywords:** payday lending, debt, health, premature death, regulation

## Abstract

Relationships between debt and poor health are worrisome as access to expensive credit expands and population health worsens along certain metrics. We focus on payday lenders as one type of expensive credit and investigate the spatial relationships between lender storefronts and premature mortality rates. We combine causes of death data from the Centers for Disease Control and Prevention (CDC) and payday lender locations at the county-level in the United States between 2000 and 2017. After accounting for county socioeconomic and demographic characteristics, the local presence of payday lenders is associated with an increased incidence risk of all-cause and specific-cause premature mortality. State regulations may attenuate these relationships, which provides insights on policy strategies to mitigate health impacts.

## Introduction

Adverse health conditions such as hypertension, cardiovascular disease, and declines in mental health are associated with debt burdens from ubiquitous access to expensive credit (1–5). Such health concerns are worrisome amidst rising debt (6) and recent deterioration in U.S. life expectancies. While overall premature mortality rates have been declining over the past two decades, since 2014, premature deaths have increased for some groups primarily due to drug overdoses and suicides (7–9). Although debt burdens have been shown to contribute to poor health (2, 5) and even premature death (1, 10, 11), the mechanisms by which communities' increasing access to expensive credit impact mortality are not well understood.

One trend in the availability of expensive credit is the expansion of higher-cost financial services like payday lenders in communities across the United States. The number of these storefront locations has increased nationwide since the mid-1990s (12–16), and the debt that borrowers accumulate from these higher-cost lenders contribute to their financial difficulties such as struggling to pay bills and delaying routine medical care (12, 13). This debt may also have effects in the aggregate, such as by contributing to communities' economic distress and worsened health outcomes regardless of whether any particular resident has borrowed expensive debt. One obstacle to identifying and testing these mechanisms is limited data on the extent of communities' financial services, making it difficult to associate communities' access to expensive credit with residents' health and premature mortality. A broad literature explores the spatial nature of business locational decisions such as fast food restaurants (17, 18), blood and plasma donation clinics (19, 20), and dollar stores (21, 22) and their associations to community economic distress with implications for public health. However, similar investigations of higher-cost lenders are limited.

In the current study, we investigate whether the presence of payday lenders is associated with premature mortality and hypothesize two mechanisms for explaining these relationships: residents living in areas with a higher number of payday lenders accumulate more higher-cost debt, and a higher density of payday lenders indicates areas' economic distress. We combine novel data including causes of death from the Centers for Disease Control and Prevention (CDC) WONDER database and the locations of payday lenders from InfoGroup USA at the county level between 2000 and 2017. Results indicate that the concentration of payday lenders may matter; though, the associations are conditional on the regulatory environment that informs payday lending practices. Importantly, we find evidence that state regulations can attenuate these relationships, especially for counties with high concentrations of payday lenders. Our findings offer new vantage points regarding the impacts of payday lending regulation. Beyond reducing financial difficulties related to paying bills, affording rent, and filing for bankruptcy that have been a focus of existing research (12–16), we suggest that regulating higher-cost financial services might advance community public health and protect against premature mortality for some groups.

## The rise of consumer debt

The use and accumulation of consumer debt are often considered indicators of a person's access to financial services and their integration into the economy (23–26). Consumer debt is increasingly required to subsidize the costs of participating in today's economy (27), which is characterized by reduced collective bargaining power (28–31), low and stagnant wages (32, 33), and widening inequality (34, 35). People rely on debt to cope with these economic trends, as indicated by steadily rising debt burdens from medical expenses, student loans, credit cards, and payday and installment loans (6). Consumer debt increased in the years following the Great Recession and reached $14 trillion in 2019 (6).

Consumer debt is also an area of stratification, where heterogeneity in the quality and cost of certain types of debt may indicate exploitation or exclusion from the economy as opposed to integration (24). Debt from higher-cost, lower quality or “alternative” financial services—such as payday and installment lenders, auto title lenders, and tax refund and anticipation lenders—is expanding, growing by about 6% each year and reaching $141 billion in 2016 (36). The alternative financial services industry has expanded with the advent of online lending, and payday loans in particular comprised $14 billion of all online lending in 2016 (26–38). State regulations that restrict or prohibit certain usurious financial services appear to effectively constrain online lenders from crossing physical geographic boundaries (39–41). As such, increases in online lending may indicate a reinforcement of the industry's spatial ties to economically distressed communities where these lenders' storefronts are disproportionately located, and allude to concerning trends in the rise of consumer debt (16, 23).

The existing literature on debt typically focuses on individual borrowing behavior (see Borck et al. (42), O'Neill et al. (43), and Simone and Walks (44) for exceptions). This includes people's borrowing from the alternative financial services industry (45–48) and the potential consequences to their finances and health (1–5, 12, 13, 49–51). Yet the rising debt burdens of individuals (5) may also accumulate to produce effects that are observable at ecological or community levels, particularly given the extent to which lending and borrowing are spatially arranged (16, 52). For instance, people are more likely to borrow, and to borrow more often, when they live in areas with an increasing concentration of alternative financial services storefronts such as payday lenders (53).

The payday loan is a specific type of higher-cost credit product among the suite of alternative financial services. Payday loans have finance fees and an average annual interest rate of about 400%, which often prevent borrowers from repaying their original loans in full. Fifteen percent of borrowers renew their loans more than 10 times (47, 52). People who borrow payday loans are often younger, between the ages of 25 and 44, and have lower levels of education and income (46, 47). There is evidence of structural racism in borrowing (54), which contributes to Black Americans being more likely to borrow relative to White Americans, all else equal (46, 55). Borrowers of higher-cost debt report using payday loans to afford routine or recurring expenses (47), and experience financial difficulties related to paying bills, affording rent, filing for bankruptcy, and receiving routine medical care (12, 13, 49–51).

## The locations of payday lender storefronts

A set of mutually reinforcing policies and practices have created spatial arrangements whereby higher-cost, lower-quality financial services are expanding and disproportionately locating in economically distressed and racially marginalized communities (15, 16, 56–64). Examining the locations of payday lenders in Colorado in 2007, a year when the state passed new legislation regulating payday loans, Gallmeyer and Roberts found that payday lender storefronts were disproportionately concentrated in census block groups with lower median incomes and higher poverty rates (62). Alternative financial services concentrate in White communities that are poor and economically distressed; though, unlike in predominantly White communities, these lenders' presence remains constant in Black communities regardless of economic indicators like income and poverty (16). Black and Latino communities have nearly twice the number of alternative financial services than do White communities (58, 60)—disparities that are amplified by segregation (16). Notably, the places where payday lenders concentrate could be the same places abandoned by other resources such as grocery stores and hospitals, making residents more susceptible to health-related concerns. In other words, a higher concentration of payday lenders could dissuade the types of development activities that have the potential to improve public health outcomes and enable economic distress, although these potential connections have yet to be evaluated.

The alternative financial services industry's expansion, and growth in payday lender storefronts in particular, has happened more rapidly in some years and in some communities than in others. For example, the number of alternative financial services storefronts increased nearly five-fold nationally between the mid-1980s and -1990s (15), before continuing to grow at an annual rate of 15% (36, 59). The notable growth in storefronts experienced by some communities coincided with the Great Recession in the mid- to late-2000s and the continued rise in consumer debt (58, 63, 64). Check cashers in New York City capitalized on the foreclosure crisis by opening new storefronts in Black and Latino communities between 2006 and 2011 (58). In California, Michigan, Ohio, and Tennessee, the number of new payday lender storefronts peaked between approximately 2006 and 2008, before leveling off in some places (64–68). Michigan's payday lender storefronts initially concentrated their expansion within the state's most populous counties during the early 2000s. Lenders deepened their presence and broadened to other counties across the state after 2005, with notable increases in counties' storefront densities occurring in 2009 and 2013 (67).

State regulation plays a role in where payday lenders are located. Given concerns that payday loans trap borrowers in cycles of debt and worsen their financial difficulties (12, 13, 15), some states have moved to regulate the industry in order to protect their residents. Payday lending densities tend to be lower in states that have strong regulations, including interest rate caps, whereas densities are relatively higher in states with permissive regulations (69). Six states and the District of Columbia currently prohibit payday lending of any kind, while 21 states do not regulate payday lending at all. In the remainder of states, regulation varies between permissive and restrictive with more restrictive regulations capping annual interest rates, preventing rollover or repeat borrowing, and assessing borrowers' ability to repay loans (70–72).

## Theoretical mechanisms linking payday lenders to mortality

There are several hypothesized mechanisms through which access to the higher-cost, lower-quality debt made available by the alternative financial services industry may influence premature mortality. One mechanism may operate at the individual level through accumulated debt. Individuals living in communities with higher concentrations of payday lenders tend to use these services at higher rates (12, 53, 73), contributing to their accumulated debt burdens and financial difficulties (13, 48, 50, 51, 74). For example, Friedline and Kepple find that individuals' increased use of alternative financial services is associated with more dense concentrations of higher-cost storefronts in their communities (53). In other words, residents who live in communities with higher concentrations of payday lenders may accumulate more debt. Among people who borrow, the financial burdens of their debt, which is an enduring source of stress that can compound over the life course (4), may place strains on their health and contribute to premature mortality. Higher-cost, lower-quality debt is associated with a range of health effects with implications for premature mortality including weight gain, depression, and suicide (2, 4, 5, 75, 76). Individuals who have accumulated debt such as from payday lenders are more likely to experience negative health consequences, including cardiovascular disease and premature mortality (1–5). In examining debt as a mediator of physical health disparities, Batomen and colleagues find that individuals with the highest amounts of unsecured debt, such as that from payday lenders, experienced an increased risk of premature death due to hypertension and cardiovascular disease, compared to their counterparts with the lowest amounts of unsecured debt (1). Taken together, these findings suggest that the presence of higher-cost, lower-quality alternative financial services like payday lenders in a person's community, as well as the debts that borrowers accumulate from these services, could contribute to rates of premature death.

Another explanatory mechanism may operate as an emergent effect (53, 77–81) and affect all residents in a community regardless of whether or not they borrow payday loans. From one perspective, the presence and or concentration of alternative financial services within a community may be a proxy for economic distress. Residents' longevity may be compromised by the extent to which the presence and or concentration of payday lenders indicate communities' economic marginalization and distress. In Toronto, Canada, a neighborhood's higher density of check cashing storefronts, which served as a proxy for economic distress, was associated with residents' increased risk of premature death (81). In a longitudinal study examining associations between county-level economic distress as indicated by unemployment rates and subprime credit ratings and mortality rates, counties that experienced the greatest distress in 2000 and 2010 had significantly higher baseline mortality rates and rates of increase (79).

Economic distress may also be causally linked to premature mortality. From this perspective, the presence and or concentration of alternative financial services is not simply a proxy for economic distress. An increase in the concentration of payday lenders may subsequently increase a community's economic distress and therefore drive up premature mortality (82–84). Existing research implies a potential causal relationship between economic distress and premature mortality (79–81) and suggests that the concentration of alternative financial services influences community economic distress (62, 78), even if these relationships are not tested directly.

Prior ecological research finds supportive evidence for effects to emerge at the community level (79, 85–87). Higher concentrations of nuisance establishments like bars and alcohol outlets that often indicate economic marginalization and distress are associated with higher rates of child abuse and neglect, a relationship hypothesized to operate through community-level mechanisms (88–91). Relationships between the spatial arrangements of marijuana dispensaries and communities' crime rates are also hypothesized to operate through community-level mechanisms (92–94). Similar relationships exist between communities' payday lender storefronts and crime rates (80, 95). Along these lines, it is plausible that individuals' increased payday loan debts contribute to premature mortality, and that lenders' presence impacts premature mortality rates vis-à-vis economic marginalization and distress.

## The current study

Using national county level data between 2000 and 2017, we examine how changes over time in the concentration of payday lender storefronts are associated with all-cause premature mortality. Among middle-aged Americans, ages 25–64, all-cause mortality rates were declining in 2000, plateaued by 2010, and began to increase after 2010 (8). These trends were especially pronounced from 2010 to 2017 when age-adjusted mortality rates increased by 6% primarily due to a substantial increase in drug overdoses, suicides, and alcoholic liver disease (8). Since most of these premature deaths are highly preventable, it is imperative to identify factors that exacerbate these deaths (81). Our analysis sheds light on two potential mechanisms that may lead to preventable premature deaths. We hypothesize that residents living in counties with higher concentrations of payday lenders have debt burdens that place strains on their health. We also hypothesize that payday lenders themselves may be a proxy of, and potential contributor to, community economic distress, which may worsen community public health outcomes. Our study cannot fully disentangle these mechanisms, but evidence of associations between payday lender presence and premature mortality will offer new pathways for scholarship on debt, access to financial services, and health. Further, a national perspective enables an evaluation of how state-level regulatory environments may impact the relationship between payday lender presence and premature deaths. For example, strong regulations that improve the affordability of payday loan products, such as capped interest rates and fees, limits on loan rollovers, or extensions of time to repayment, may subsequently attenuate any positive relationship.

## Data

We combine data from several sources to develop a novel dataset for this study. First, we obtain historical data on payday lender storefront locations in the United States between 2000 and 2017 from InfoGroup. These data include the address, business name, and annual operating status for every payday lending storefront in the United States. We generate an annual file of active payday lenders using Standard Industry Classification business codes and word searches within company names (e.g., “cash advance”, “payday”). We then match geocoded business addresses to county boundaries to generate a county-level data file that captures the number of active storefronts in each county and each year. We then bring in data on premature mortality at the county level using data from the Center for Disease Control and Prevention's (CDC) WONDER database (96). We also include county-level socio-economic and demographic information using data from the Census and American Community Survey (97). Finally, we include annual data on state-level payday lending regulations from the National Conference of State Legislatures (72).

## Key measures

Our outcome of interest is derived from the count of *premature deaths* in each county in each year. We define premature deaths as deaths from any cause among 20–59 year-olds, following the approach used by Matheson et al. (81). The CDC suppresses mortality counts between 0 and 9 and considers rates that use counts below 20 deaths to be unreliable. As such, we restrict our analytic sample to counties that have 20 or more premature deaths in a given year. Of the 3,134 eligible counties in the United States, 2,626 meet this criteria for at least 1 year between 2000 and 2017. In secondary models, we also evaluate cause-specific premature deaths for cardiac-, mental health- and assault-related deaths^1^. These models use subsets of counties that have non-suppressed counts of these deaths and seek to provide additional insights on possible individual- and community-level mechanisms linking payday lender presence to premature deaths.

Our key variable of interest is a three-level categorical measure of *payday lender presence*. The reference group is 0 lenders within a county, which we compare to counties that have 1–3 payday lenders, and those that have 4 or more. We base these categories on the average numbers of alternative financial services storefronts found in previous research (16, 60)^2^.

A second variable of interest is a constructed measure of *regulatory strength*. This measure refers to the strength of each state's payday lending regulatory environment in a given year. We use a four category measure. The reference group is states with *no regulations*, which is compared to states with *weak regulations, moderate regulations*, and *strong regulations*. A weak regulatory environment is defined as one where the state has a law on the books requiring payday lending licensing and registration. A moderate regulatory environment refers to those that limit rollovers or require lower interest rates. A strong regulatory environment refers to states that have fully prohibited payday lending or have strict interest rate caps set to 36% APR. For our analysis, we include all states, including those that prohibit payday lending. Supplemental models using only states that allow payday lenders produce similar results.

We include several time-varying control variables to better isolate the relationship between local payday lending environments and mortality outcomes. We include continuous measures of the county's *share of poverty, share of male residents, share of Black and Latino residents, share of new residents moving into the county in the prior year*, and *share of urban residents* in a given year. Covariates for race, sex assigned at birth, poverty, and urbanicity are standard controls included in analyses on payday lending [see, e.g., Faber (16)]. We additionally include the measure of county residential mobility as a proxy for duration of exposure to the county environment.

Variation in the population at risk of premature death across counties is accounted for using the *population of individuals ages 20–59* as an exposure term in our models, which converts our count of premature deaths to a rate. All measures come from the Census and the American Community Survey (ACS) (97). We create annual measures for years 2008–2017 using the five-year ACS data with the year of interest as the midpoint. For years 2001 - 2007, we use linear interpolation between the 2000 Census and the 2006–2010 ACS (where 2008 is the midpoint) to generate annual estimates.

Table 1 presents descriptive statistics for all variables used in analysis. The premature mortality count for a typical county is 191 but with substantial variation in these counts, which range from 20 in some counties to well over 5,000 annually in large urban counties like Cook County, IL and Los Angeles County, CA. In our data, 38% of county-years have no payday lenders, about 27% have one to three payday lenders, and about 35% have four or more. In this latter category, it is not uncommon for a county to have numerous lenders in a given year; close to 37% of counties in this category (13% of the overall sample) have 10 or more lenders in at least 1 year. Regulatory environments are also mixed; for example, 47% of county observations are in weak state regulatory environments and 24% are in strong state regulatory environments.

**Table 1:** Descriptive statistics.

	**Mean (SD) or Proportion**
*Outcome Variable*	
County Premature Mortality (count)	191.27 (483.28)
*County Lender Composition*	
0 Lenders in County	0.38
1–3 Lenders in County	0.27
4+ Lenders in County	0.35
*State Regulatory Strength (share of county-year observations)*	
No Regulations	0.21
Weak Regulations	0.47
Moderate Regulations	0.07
Strong Regulations	0.24
*Covariates*	
Population Ages 20-59	61,413 (177,369)
Share of Male Residents	0.49
Share of Residents in Poverty	0.15
Share of Black/Latinx Residents	0.18
Share of Movers in the Past Year	0.07
Share of Urban Residents	0.49
*n*	42,230

2,626 counties contributed 42,230 observations over the analytic period. Covariate statistics refer to the underlying continuous measures that were used to create the categorical measures used in models.

## Empirical strategy

We first present descriptive associations between the local payday lending environment and premature deaths. Because our outcome of interest is a count variable across space and time, we next fit longitudinal Poisson regressions with random effects (98). Inclusion of the exposure term, population aged 20–59, adjusts the model results to reflect incidence risk of premature deaths at the county level (i.e., converts the count to a rate). We report incidence risk ratios^3^, and standard errors are clustered at the county level in all models. All models include state and year fixed effects to account for omitted variables that vary by state and year.

We proceed in two stages. First, we model the association between payday lender presence and premature death. In this analysis, results from our first Poisson regression (M1) provide estimates from a model of the change in incidence risk of premature deaths as a function of lender presence, net of state and year fixed effects. M2 adds our set of time-varying county-level controls. M3 presents results from a model that interacts payday lender presence with all covariates. We include an interacted model to underscore that associations between payday lender presence and premature death rates may be conditional on other community characteristics, given known demographic and socio-economic disparities in both premature deaths and payday lender locations.

Second, we evaluate how payday lending regulation moderates the relationship between lender density and premature deaths, first with a model interacting lender presence and regulatory strength net of state and year fixed effects (M4), and then adding the set of time varying controls (M5). This set of models provides insights on whether and how regulation of high-cost lending may reduce premature mortality. For ease of interpretation, we do not interact these models with the demographic and socio-economic covariates.

## Limitations

Our analysis is not without limitations. Data limitations include suppression of counties with < 20 mortality counts. Main models are not spatially weighted to account for geographic clustering of premature death and payday lender counts due to modeling limitations (see Appendix A for discussion). Although we hypothesized individual- and community-level mechanisms to explain higher-cost lenders' effects on premature death, data limitations make direct tests of these mechanisms suggestive. Future research should attempt to elucidate these explanatory mechanisms, particularly how and the extent to which the concentration of payday lenders represents economic distress, encourages economic distress by dissuading other types of development activities, and contributes to poor public health outcomes. Moreover, payday lenders have not been present for very long by our first year of data, meaning that exposure to lenders could be limited and our data do not fully capture cumulative effects. Any associations that are suggestive of cumulative effects may be underestimated. Finally, we look at overall premature death counts and do not examine the effects of payday lender presence on premature deaths by subgroup (e.g., by sex assigned at birth and race/ethnicity), and future research should examine the potential heterogeneity in effects across subgroups.

## Results

### Descriptive maps of key variables

**Figure 1:**
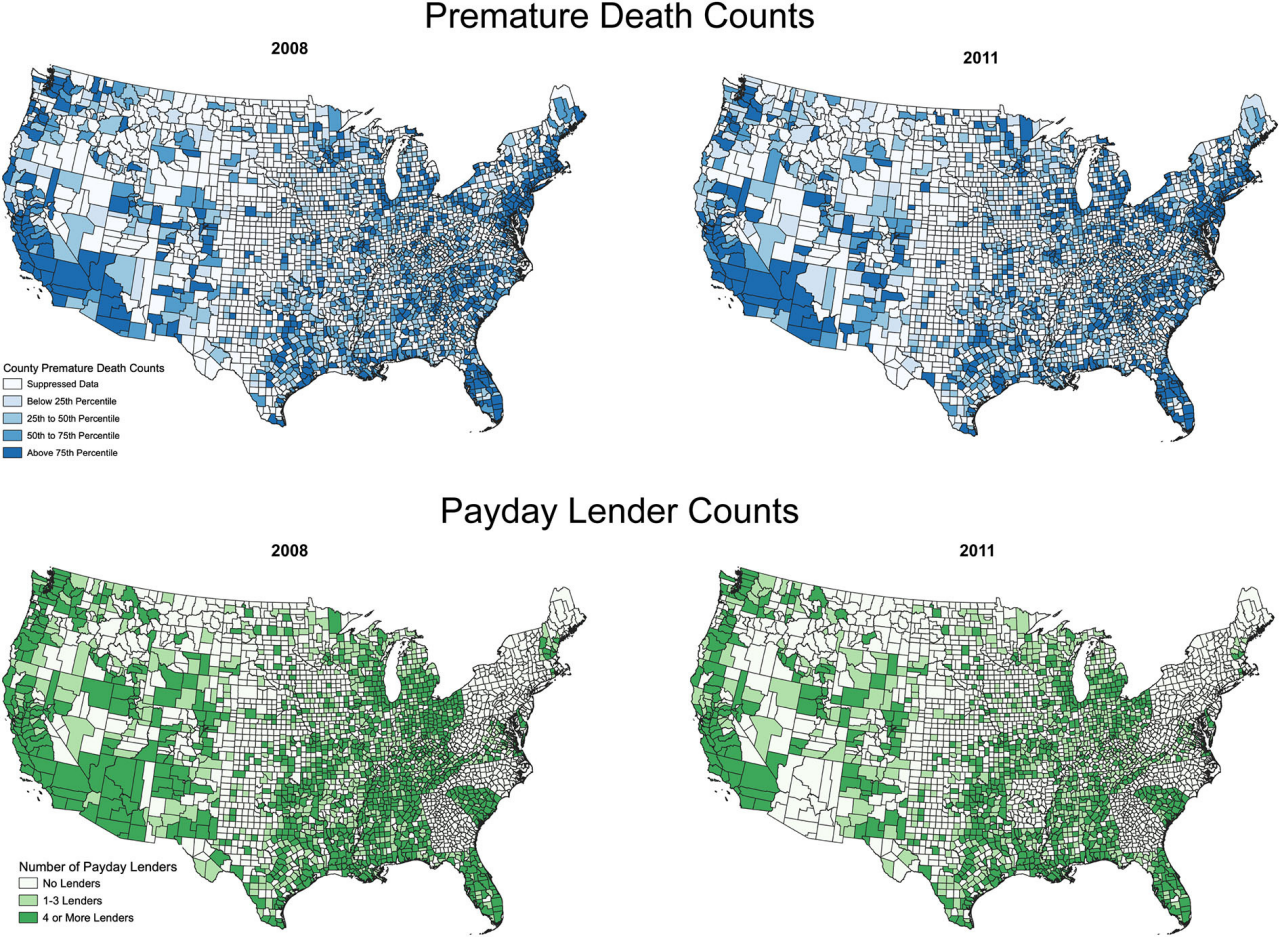
Counts of premature deaths and payday lenders over time (by county and year) for counties in the continental United States. Maps created using QGIS software, county boundaries reflect 2010 Census boundaries.

Figure 1 presents counts of premature deaths and counts of payday lenders for 2 years in our analysis, 2008 and 2011, using all available data from the CDC and InfoGroup. These years correspond with the start and end of the Great Recession, which impacted both premature death rates (99) and use of payday loans (100). The top panel, which features county-level counts of premature deaths from all causes, shows a consistent clustering of premature deaths in the South, with more counties experiencing premature counts in the 75th percentile or higher in 2011. It also shows that the majority of the suppressed CDC data is largely from rural counties in the central and western United States; these counties will be excluded from our analyses.

The bottom panel, which features counts of payday lenders, shows that the count of counties with four or more lenders (top categories) was strongest in 2008, with fewer counties reporting 4 or more payday lenders in 2011. Much of the decline by 2011 is likely due to regulatory interventions that several states enacted, rather than a decline in demand. Use of payday loans increased during the Great Recession (100), but payday lenders also faced increased oversight in several states, which has an impact on where they operate (101). Many of the counties that have suppressed CDC data also have few payday lenders (e.g., central United States). Beyond that, the two panels suggest positive associations between premature deaths and payday lenders; many of the counties with relatively high counts of premature deaths also have four or more payday lenders (see, e.g., parts of Texas and the Florida panhandle). These descriptive correlations also reflect other factors like urbanicity, which will be accounted for in our modeling.

### Spatial autocorrelation of variables

Our models do not account for spatial clustering of premature deaths and payday lenders. Given the spatial clustering observed in Figure 1, we assessed the spatial autocorrelation of the dependent and independent variables for the inclusion of spatial weights in our models using Moran's I. There is spatial autocorrelation for our measures of payday lender counts and premature deaths in each year of our analysis (*p* < 0.001). In appendix A, we compare our main results to those from spatially weighted OLS models, which are less ideal for a count outcome but permit an evaluation of potential spatial spillover effects among neighboring counties. The spatially weighted models indicate that spatial autocorrelation does impact results; the models presented below are likely underestimating the effects of payday lender presence on premature mortality.

### Association between payday lender presence and all-cause premature mortality

Table 2 presents the predicted incidence risk ratios adjusted for state and year fixed effects (M1) and incidence risk ratios that are also adjusted for controls (M2) and interactions with controls (M3). Full model results are available in Appendix B. Model 1, which includes no controls, shows a significant, positive association between counties with four or more lenders and the risk of premature deaths, with a 2% increase in risk of premature death over counties without lenders (RR = 1.020, *p* < 0.05). Counties with 1–3 lenders have little difference in risk of premature death compared to counties without lenders (RR = 1.009, *p* > 0.05). After adjusting for controls, M2 shows that the adjusted risk ratio continues to be significant and positive for counties with 4+ lenders compared to counties without lenders (RR = 1.021, *p* < 0.05). Appendix B also confirms that results for covariates in M2 have directions that are largely in line with expectations. For example, an increase in the share of urban residents is associated with a reduction in risk of premature mortality (*p* < 0.05), in line with prior research that shows that premature deaths are higher in rural areas (89). Further, an increase in the share of mobility in the county is significantly associated with a reduced risk of premature death; other covariates' coefficients have suggestive directions and most are significant (e.g., as the share of male residents increases, there is an association with a slightly elevated risk of premature death, in line with work showing differences in sex assigned at birth in premature deaths (*p* < 0.05).

**Table 2:** Unadjusted and adjusted incidence risk ratios for premature deaths, by lender presence.

	**M1**	**M2**	**M3**
No Lenders (reference)	1	1	1
1–3 Lenders	1.009 (0.007)	1.011 (0.006)	1.023^*^ (0.006)
4+ Lenders	1.020^*^ (0.010)	1.021^*^ (0.008)	1.017^*^ (0.007)
Control Variables		Y	Y
Interactions with Controls			Y
State FEs	Y	Y	Y
Year FES	Y	Y	Y
*N*	42,230 county-years; 2,626 counties	

Incidence risk ratios relative to counties with no lenders; robust standard errors in parentheses;

* *p* < 0.05. Exposure term is population ages 20–59. Derived from Models 1–3 in Appendix B using Stata margins and nlcom commands.

**Figure 2:**
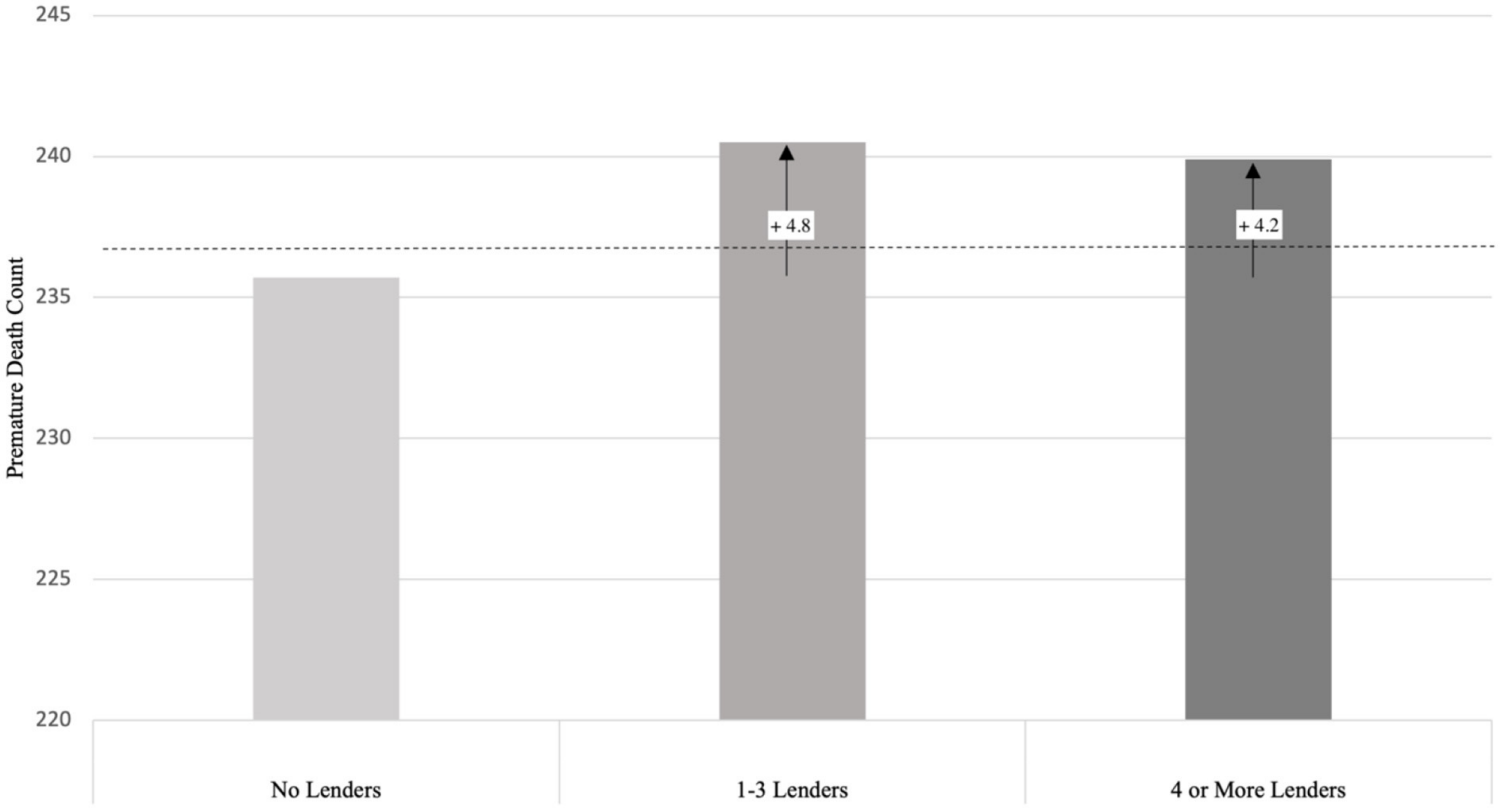
Predicted excess premature deaths in counties with lenders. The higher adjusted incidence risk ratios (*p* < 0.05) in M3 of Table 2 for counties with 1 to 3 and 4 or more lenders translate to 4.8 and 4.2 excess deaths, respectively, compared to counties with no lenders. Estimated death counts are derived from Model 3 in Appendix B using Stata *margins* and *lincom* commands. Dashed line represents overall grand mean (238.4 deaths), derived from same model. 2,626 counties contributed 42,230 observations.

When the model is interacted (M3), the main effects for counties with 1–3 lenders and 4 or more lenders are significant (*p* < 0.05), as are some interaction effects with county socio-economic and demographic covariates (see Appendix B). As shown in Table 2, counties with 1–3 lenders have a 2.3% increase in risk of premature mortality, and counties with 4 or more lenders have a 1.7% increase in risk, compared to counties without lenders (*p* < 0.05 for both). To put this in context, Figure 2 presents predicted premature mortality counts by county type. Counties with no lenders have an average predicted premature mortality count of 235.7 deaths when all covariates are at their means. Compared to counties without lenders, the presence of 1–3 lenders was associated with a predicted excess of 4.8 deaths and the presence of 4 or more lenders was associated with a predicted excess of 4.2 deaths^4^.

### Associations between payday lender presence and cause-specific premature mortality

Though data are more limited, we also evaluate three cause-specific rates of premature mortality, evaluating deaths that stem from mental and behavioral health disorders, cardiac issues, and assaults. Deaths due to mental health (e.g., suicides) in communities with higher-cost lenders may speak to more immediate individual-level mechanisms where stress related to increased debt burdens is linked to premature deaths, given known literature that finds debt can negatively impact mental health [e.g., Fowler et al. (60)]. Cardiac-related deaths may provide a longer-term view of the health consequences of the accumulation of personal debt over time [e.g., Batomen et al. (1), Eisenberg-Guyot et a. (2), Nelson et al. (3), Sweet et al. (4), and Fitch et al. (5)]. Deaths due to assault may speak to broader community-level factors, where the presence of payday lenders as an indicator of economic distress leads to higher risks of these deaths. We know from prior work that the presence of payday lenders has been linked to increased violent crime in local areas [e.g., Kubrin et al. (80)], and assault-related deaths may speak to the more immediate ecological impacts of payday lender presence.

The number of counties with non-suppressed information on these deaths is lower, and thus these analyses are more limited. We evaluate mental health and cardiac related deaths using a shared sample of 1,213 counties with 11,519 observations. We separately evaluate assault related deaths using an even smaller sample of 193 counties and 2,184 observations; this cause of death is rarer and few counties have sufficient counts of deaths to be included in analysis. For the analysis of assault related deaths, we reduce our measure of lender presence to two categories: no lenders, or any lenders: this is because the vast majority of counties with lenders have 4 or more locations (only five counties had one to three lenders). Models for these disaggregated analyses mirror Table 2 using the same covariates and interactions. The smaller sample sizes reduce the precision of estimates, and we caution that these results reflect a select set of counties that may not be nationally representative.

Results in Table 3A show strong evidence of a positive association between payday lender presence and mental-health related deaths. In the model for mental-health related deaths net of covariates (M2), having 1–3 lenders or 4 or more lenders is associated with a substantially higher risk of premature mortality compared to counties with no lenders (RR = 1.178, *p* < 0.01 and RR = 1.167, *p* < 0.05). When interacted with county covariates, the adjusted risks remain elevated, although with large confidence intervals (RR = 1.082, *p* > 0.05, and RR = 1.068, *p* > 0.05, respectively). In these same counties, we see more modest evidence of connections to cardiac-related deaths (M4 - M6). Having 1–3 lenders is positively associated with a higher risk of cardiac-related premature mortality compared to having no lenders; however, the results are only significant for those counties with 1–3 lenders in Model 5 (RR = 1.054, *p* < 0.05). Finally, as shown in Table 3B, in the more limited set of counties for premature deaths due to assaults (M3), there are positive associations between having any lenders in a county compared to no lenders, with magnitudes similar to that for the risk of mental-health related premature deaths (e.g., RR = 1.077, *p* > 0.05 in M3 of Table 3B).

**Table 3A:** Incidence risk ratios for premature deaths from mental health and cardiac-related causes.

	**Mental Health**			**Cardiac**		
	**M1**	**M2**	**M3**	**M4**	**M5**	**M6**
No Lenders (reference)	1	1	1	1	1	1
1–3 Lenders	1.184^**^ (0.076)	1.178^**^ (0.071)	1.082 (0.056)	1.047 (0.024)	1.054^*^ (0.023)	1.043 (0.028)
4+ Lenders	1.139 (0.083)	1.167^*^ (0.079)	1.068 (0.055)	0.984 (0.020)	1.014 (0.018)	0.976 (0.022)
Control Variables		Y	Y		Y	Y
Interactions with Controls			Y			Y
State FEs	Y	Y	Y	Y	Y	Y
Year FES	Y	Y	Y	Y	Y	Y
*N*	11,519 county-years; 1,213 counties					

Incidence risk ratios relative to counties with no lenders; robust standard errors in parentheses. Exposure term is population ages 20–59.

* p < 0.05;

** p < 0.01. Full models available upon request.

**Table 3B:** 

	**Assault**		
	**M1**	**M2**	**M3**
No Lenders (reference)	1	1	1
1 or More Lenders	1.220^**^ (0.091)	1.193^*^ (0.095)	1.077 (0.071)
Control Variables		Y	Y
Interactions with Controls			Y
State FEs	Y	Y	Y
Year FES	Y	Y	Y
*N*	2,184 county-years; 193 counties		

Incidence risk ratios relative to counties with no lenders; robust standard errors in parentheses. Exposure term is population ages 20–59.

* p < 0.05;

** p < 0.01. Lender categories collapsed into two groups due to small sample of 1–3 lender counties in this sub-analysis. Full models available upon request.

Together, these results show initial support for both individual- and community-level mechanisms, with some indication that the public health impacts of payday lender presence may be more immediate as shown by the large magnitudes of the relative risks for mental health and assault related deaths in counties with lenders. The comparatively more modest associations between cardiac deaths and payday lender presence may be due to the fact that the full cumulative effects of local industry presence on public health have not been realized. In our analytic period, payday lending is relatively new; most storefronts started opening nationwide in the early 2000s, and connections to longer term health issues may not be known for some time. Additional analyses (not shown) of all-cause mortality that include a control for state-level credit card debt per capita find that this control has a significant, positive association with premature deaths; though, it does not meaningfully change the payday lender—premature death association. This further suggests that the mechanisms behind the relationship may extend beyond individual-level debt burdens. These analyses remain suggestive; more research is needed to fully understand mechanisms behind the association between communities' payday lender presence and premature deaths.

### Impact of regulatory interventions on relationship of interest

We return to our main analysis of all-cause premature mortality and include an interaction between payday lender presence and regulatory strength to understand whether any relationship between lender presence and premature deaths may be dependent upon the regulatory environment. Table 4 presents results of this lender presence by regulation interaction with just state and year fixed effects (M1) and results from a model that also controls for other covariates (M2). These results indicate that the impacts of payday lender presence are conditional on the regulatory context. Full model results are available in Appendix C.

**Table 4:** Incidence risk ratios for premature deaths by lender presence and regulatory strength.

	**M1**	**M2**
No Lenders, No Regulations (references)	1	1
No Lenders, Weak Regulations	1.111^***^ (0.012)	1.086^***^ (0.012)
No Lenders, Moderate Regulations	1.160^***^ (0.023)	1.103^***^ (0.024)
No Lenders, Strong Regulations	1.071^***^ (0.016)	1.047^***^ (0.014)
1–3 Lenders, No Regulations	1.010 (0.008)	1.003 (0.007)
1–3 Lenders, Weak Regulations	1.118^***^ (0.013)	1.093^***^ (0.013)
1–3 Lenders, Moderate Regulations	1.143^***^ (0.024)	1.108^***^ (0.022)
1–3 Lenders, Strong Regulations	1.068^***^ (0.016)	1.052^***^ (0.014)
4+ Lenders, No Regulations	1.078^***^ (0.016)	1.056^***^ (0.012)
4+ Lenders, Weak Regulations	1.107^***^ (0.010)	1.087^***^ (0.016)
4+ Lenders, Moderate Regulations	1.086^***^ (0.021)	1.071^***^ (0.020)
4+ Lenders, Strong Regulations	1.082^***^ (0.019)	1.054** (0.017)
*N*	42,230 county-years; 2,626 counties	

Incidence risk ratios relative to counties with no lenders within states with no regulations; robust standard errors in parentheses;

*** *p* < 0.001. Exposure term is population ages 20−59. Derived from Models 1 and 2 in Appendix C using Stata *margins* and *nlcom* commands.

There are significant differences that are large in magnitude for premature death—payday lender associations across regulatory contexts. M2 shows that, relative to counties with no lenders in states without regulations, every other combination of regulatory environment and lender presence has a significantly higher risk of premature mortality. The only exception is among counties with 1–3 lenders in states without regulation, which remains marginally higher (RR = 1.003, *p* > 0.05). In weak and moderate regulatory environments, relative risks range between 1.07 and 1.11, or seven to 11% higher, compared to counties with no lenders and no regulations, while relative risks tend to be lower in strong regulatory environments (around 1.05 for each county type, or 5% higher). These patterns suggest that the variation in the degree of attenuation that regulation can achieve depends on its strength. For example, counties with 1–3 lenders move from a relative risk of 1.09 in a context of weak regulations to 1.11 in a context of moderate regulations; in other words, the risk of premature death actually increases by 2% when moving from weak to moderate regulations. However, the shift from moderate to strong regulations for these counties results in a *reduction in risk* by 6% (moving from 1.11 to 1.05). This suggests that strong regulations do comparatively better in dampening the risk of premature mortality compared to weak and moderate regulations, which actually experience increases in risk compared to even less regulation.

That these patterns are true irrespective of the number of lenders is somewhat puzzling. Similar patterns occur for counties that have no lenders across these regulatory contexts, and we would expect regulation to impact counties with lenders but have little effect on counties without. This might be due in part to compositional changes that occur when regulation takes effect. For example, when a state shifts from moderate to strong regulations, there are some counties where all lenders leave, as shown by prior work (101–103). When this occurs, the county would get reclassified as part of the “no lenders, strong regulations” group in our models. The average adjusted risk of 1.05 for this group might be picking up some of the higher initial risk of counties that had been in the 1–3 or 4+ lender categories under a moderate regulatory context. More research is needed to fully understand why regulation impacts the public health of counties without lenders.

**Figure 3:**
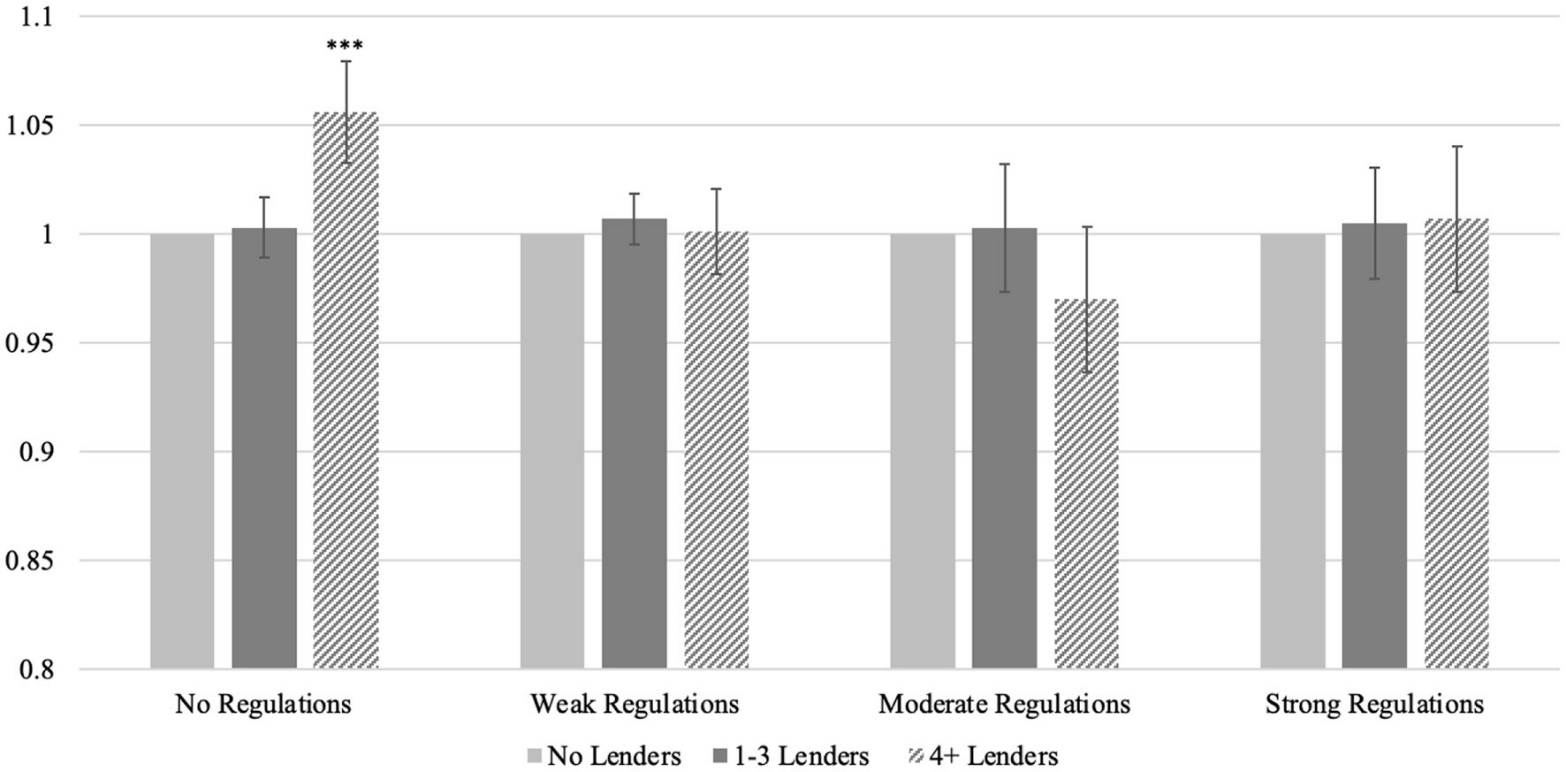
Within-regulatory group relative risks for premature deaths. Within-period incidence risk ratios relative to counties with no lenders (left-most column in each group); error bars reflect 95% confidence intervals. ***p < 0.001, compared to counties with no lenders. Exposure term is population ages 20–59; 2,626 counties contributed 42,230 observations. Derived from Model 2 in Appendix C using Stata *margins* and *nlcom* commands.

Figure 3 displays estimated relative risks *within* regulatory context, derived from Model 2. This figure does not show increases in levels relative to one common reference group, as shown in Table 4, but rather compares across county lender categories within the same regulatory environment. In the absence of regulation, the relative risk of premature deaths is the highest for counties with four or more lenders (RR = 1.06, *p* < 0.001), and then differences between high concentration counties and those with zero and 1–3 lenders diminish as regulations strengthen. In moderately regulated environments, the risk for counties with four or more lenders is marginally lower relative to that of counties without lenders (RR = 0.97, *p* > 0.05). Relative risks return to parity under strong regulatory environments, all else equal. This within-context comparison underscores that the lack of regulation impacts public health in counties with high concentrations of lenders the most, and that these counties gain relatively greater public health benefits under contexts with enhanced regulation.

## Concluding discussion

The relationship between high debt burdens and poor health raises concerns about the expansion of the payday lending industry, which sells expensive loans that are hard to repay (47, 52) and contribute to borrowers' financial difficulties (12, 13). Robust literatures explore the relationships between payday lenders and financial difficulties [e.g., (12, 77)] and high debt burdens and poor health [e.g., Batomen et al. (1) and Eisenberg-Guyot et al. (2)], laying the groundwork to connect these lines of inquiry. Importantly, as the number of these industry storefronts expanded, some states began trying to protect borrowers by placing new restrictions on payday lenders such as capping usurious interest rates and preventing the renewal or re-borrowing of these loans, which may have public health benefits. We explore these associations spatially at the county level and our findings, described below, offer new pathways for inquiries into payday lending and effective regulation.

Several key findings elucidate any relationships between the distribution of payday lenders and community health. First, we find that after accounting for socioeconomic covariates, the risk of all-cause premature death is significantly higher in counties with four or more payday lenders and one to three lenders, relative to counties without. Secondary analyses that disaggregate causes of premature deaths lend some support for both proposed mechanisms for this association. Higher risks of mental health related deaths and modestly higher risks of cardiac related deaths suggest that residents' longevity in these communities may be compromised by individual-level stress due to increased debt burdens. Higher risks of assault related deaths suggest that exposure to heightened community economic distress as proxied by payday lender presence may also compromise longevity. Because our analysis remains at the county level, it is not possible to fully disentangle community- and individual-level mechanisms; though, taken together, the all-cause and specific cause analyses underscore that payday lender presence is associated with poorer community health, even after accounting for community demographic composition, poverty, and urbanicity.

Second, we find evidence of moderating effects of regulation, whereby the influence of a county's concentration of payday lenders on the risk of all-cause premature death is conditional on regulatory context. These findings provide evidence that better regulations may have beneficial public health impacts in areas with a relatively large number of lenders. This modest attenuation lends some support to the notion that improved regulation may have positive spillover effects on community health. These findings are notable because they allude to the importance of broadening policy conversations on financial regulation to include the effects on social, physical, and mental well-being. Depending on the extent to which the effectiveness of regulation is evaluated in economic terms, current policy conversations may underestimate the economic benefits of regulation by focusing primarily on financial difficulties and well-being.

Regulation appears to matter even for counties without lenders. This puzzling finding could be explained by the extent to which regulations targeting payday lenders also discourage or supplant other types of storefronts and businesses that contribute to a county's economic marginalization and distress. Similar to regulation's positive spillover effects on community health, perhaps there are also spillover effects onto usurious and other predatory businesses that undermine community health even in absence of payday lenders. Future research should investigate this possibility.

In the United States, geographic inequalities in health and mortality are growing. The substantial spatial variation in mortality rates makes it important to understand the links between local built environments, policy contexts that inform those environments, and public health. For instance, the absence of grocery stores and hospitals from communities—forms of food and healthcare apartheid enabled by policy decisions and that often accompany other indicators of economic marginalization and distress—has implications for public health (104, 105). We provide evidence that the availability of expensive credit also matters, using geographic variation in the presence of payday lenders and connections to mortality. Understanding the contributions of payday and other high-cost lenders to mortality can aid in identifying potential underlying mechanisms and the possibility for regulation to attenuate their effects. We suggest that, in this context, regulation has the potential to protect against premature mortality for some groups. Future research will need to investigate these relationships in the years during and after the COVID-19 pandemic, which notably changed people's life expectancy and experiences with financial difficulties, as well as influenced business turnover and storefront locational decisions.

## Data availability statement

The data analyzed in this study is subject to the following licenses/restrictions: The Center for Disease Control and Prevention's WONDER database is available for public use (https://wonder.cdc.gov/wonder/help/ucd.html). Annual data on state-level payday lending regulations from the National Conference of State Legislatures is available for public use (https://www.ncsl.org/research/financial-services-and-commerce/payday-lending-state-statutes.aspx). Historical data on payday lender storefront locations in the United States between 2000 and 2017 is available for purchase from InfoGroup or available through some university library subscriptions, such as the University of Michigan. Requests to access these datasets should be directed to https://www.data-axle.com/contact-us/?gclid=CjwKCAjw2rmWBhB4EiwAiJ0mtSffVEq4u6aa2W9P7ScizvyA-RQXNDJt-PpXLJoMQ9EXQHzAMNBPkRoC4kgQAvD_BwE#contact_us_location_3?utm_term=data%20axle%20headquarters&amp;utm_campaign=Corporate+Brands+%7C+PR&amp;utm_source=google&amp;utm_medium=cpc&amp;hsa_acc=9152831390&amp;hsa_cam=13480869329&amp;hsa_grp=123161790413&amp;hsa_ad=529715951002&amp;hsa_src=g&amp;hsa_tgt=kwd-1307381766427&amp;hsa_kw=data%20axle%20headquarters&amp;hsa_mt=b&amp;hsa_net=adwords&amp;hsa_ver=3.

## Author contributions

All authors listed have made a substantial, direct, and intellectual contribution to the work and approved it for publication.

## Funding

MA's time for this research was supported by the T32 AG00129 grant, awarded to the Center for Demography of Health and Aging at the University of Wisconsin-Madison by the National Institute on Aging.

## Conflict of interest

The authors declare that the research was conducted in the absence of any commercial or financial relationships that could be construed as a potential conflict of interest.

## Publisher's note

All claims expressed in this article are solely those of the authors and do not necessarily represent those of their affiliated organizations, or those of the publisher, the editors and the reviewers. Any product that may be evaluated in this article, or claim that may be made by its manufacturer, is not guaranteed or endorsed by the publisher.

## Footnotes

^1^We use the CDC WONDER database to pull county-level counts of premature deaths due to mental health [IC10 codes - F01-F99 (mental and behavioral health disorders) and X60 - X84 (intentional self-harm)], cardiac [I10 - I51 (e.g., hypertension, heart disease)], and assault (X85-Y09 “assault”).

^2^Adjusting the categories to evaluate counties with 0 lenders relative to counties with 1–3, 4–10, and 11 or more lenders produces substantively similar results.

^3^With our exposure term, incidence risk is defined as the number of premature deaths divided by the population ages 20–59 (i.e., the population at risk of a premature death) for each county in each year.

^4^Calculated using results from Model 3 in Appendix B, using the margins postestimation commands in Stata. We keep covariates at their means to approximate an average U.S. county during our analytic period.
