# Supplementary material for: Payday lenders and premature mortality
Source: Front Public Health. 2022 Oct 18;10:993585. doi: 10.3389/fpubh.2022.993585 (PMC9623148; doi:10.3389/fpubh.2022.993585)
Supplement: Supplementary file 1 [file Data_Sheet_1.docx]

**Payday Lenders and Premature Mortality**

**Appendix A.** Comparison of Spatial OLS Model and Poisson Regression

Our main models (Tables 2, 3, 4, Appendices B, C) may be biased because we do not account for spatial spillover effects. Moran’s I tests that use a queen contiguous spatial weights matrix reveal that there is spatial autocorrelation for both premature deaths counts and payday lender counts across U.S. counties in each year, with geographic clustering across neighboring counties. Typically, econometric models could account for this spatial association using spatially lagged dependent and independent variables on the right-hand side of the model (Anselin 2010). This works well for models with continuous dependent variables but Poisson models are less well-suited to spatial weighting because count data like premature deaths are nonlinear (for a review see Glaser 2017).

Below we compare spatial OLS random effects regressions to non-spatial Poisson models. We consider both spatial lag OLS models that include a spatial lag for the dependent variable and independent variable of interest (payday lender presence) as well as a spatial error OLS model. The spatial lag model assumes that the premature death rates in county *i* could be impacted by lender presence in the county and in its neighboring counties. This is plausible; one could imagine that being in close proximity to a lender, even if it is technically in a neighboring county, might be associated with health outcomes. The error model corrects for spatially correlated omitted variables. The OLS models treat the count data as continuous, and we exponentiate the results to approximate adjusted risk ratios. Models include all U.S. counties. For counties where mortality rates are suppressed or unreliable (fewer than 20 deaths in a given year), we code these as having 0 deaths. This is necessary to ensure that each county has spatial neighbors in each year in the OLS models. A comparison of the adjusted risk ratios from the Poisson model in A1 below and the results for M3 in Table 2 in the main text show that the inclusion of suppressed counties in the count model does not meaningfully differ from our main results discussed in the paper.

Table A1 below shows that the adjusted risk ratios for the spatial models are significantly higher than the Poisson models. The full results in Table A2 show that the spatial lag term for payday lenders in the first OLS models is not statistically significant when all control measures are included, but the spatial lag term for the dependent variable is significant. The spatial error term in the second model is also significant. These models underscore that spatial associations across counties contribute to higher adjusted risks of premature deaths due to the way that the counts are geographically clustered. However, the treatment of count data as continuous introduces its own bias by assuming linearity. For our main analyses, we proceed with the non-spatial Poisson regressions because they are appropriate for count data, but we caution that our models may be underestimating the effects of payday lending presence on premature mortality because they do not account for spatial autocorrelation.

**References**

A1 Anselic L. Thirty years of spatial econometrics. Regional Science (2010) 89: 3-25.

A2 Glaser S. A review of spatial econometric models for count data. Hohenheim Discussion Papers in Business, Economics and Social Sciences, No. 19-2017, Universität Hohenheim, Fakultät Wirtschafts- und Sozialwissenschaften, Stuttgart. (2017) http://nbn-resolving.de/urn:nbn:de:bsz:100-opus-13975.

A1. Adjusted Risk Ratios for Spatial and Non-spatial Models

|  | Spatial Lag  OLS | Spatial Error OLS | Non-Spatial Poisson |
| --- | --- | --- | --- |
| No Lenders | 1 |  | 1 |
| 1-3 Lenders | 1.081***  (0.027) | 1.068*  (0.025) | 1.032*** (0.007) |
| 4+ Lenders | 1.061  (0.034) | 1.030  (0.027) | 1.025*** (0.009) |
| Control Variables | Y | Y | Y |
| Interaction with Controls | Y | Y | Y |
| Spatial Lags (DV, IV) | Y | -- | -- |
| Spatial Error Term | -- | Y | -- |
| N | 56,412 | 56,412 | 56,412 |

A2. Full Model Results for Spatial OLS and Non-spatial Poisson Regressions

|  | Spatial Lag | Spatial Error | Poisson |
| --- | --- | --- | --- |
|  | M1 | M2 | M3 |
| Payday Lender Presence (Ref = 0 Lenders) |  |  |  |
| 1-3 Lenders | 0.727 | 0.742 | 0.512** |
|  | (0.162) | (0.166) | (0.116) |
|  |  |  |  |
| 4 or more lenders | 1.245 | 1.290 | 0.438** |
|  | (0.424) | (0.440) | (0.123) |
| Covariates |  |  |  |
| Male Share | 0.984*** | 0.983*** | 1.019* |
|  | (0.003) | (0.003) | (0.008) |
|  |  |  |  |
| Black/Latinx Share | 1.008*** | 1.008*** | 0.998 |
|  | (0.001) | (0.001) | (0.003) |
|  |  |  |  |
| Poverty Share | 0.995*** | 0.995*** | 0.996 |
|  | (0.001) | (0.001) | (0.004) |
|  |  |  |  |
| Mobility Share | 0.991*** | 0.991*** | 0.973*** |
|  | (0.002) | (0.002) | (0.005) |
|  |  |  |  |
| Urban Share | 1.002** | 1.001* | 1.009*** |
|  | (0.001) | (0.001) | (0.001) |
| PL X Covariate Interactions |  |  |  |
| 1-3 Lenders X Male Share | 1.010* | 1.010* | 1.010* |
|  | (0.004) | (0.004) | (0.004) |
|  |  |  |  |
| 4+ Lenders X Male Share | 1.002 | 1.001 | 1.015** |
|  | (0.007) | (0.007) | (0.005) |
|  |  |  |  |
| 1-3 Lenders X Black/Latinx Share | 0.996* | 0.996* | 1.010*** |
|  | (0.002) | (0.002) | (0.003) |
|  |  |  |  |
| 4+ Lenders X Black/Latinx Share | 0.991*** | 0.991*** | 1.006 |
|  | (0.002) | (0.002) | (0.004) |
|  |  |  |  |
| 1-3 Lenders X Poverty Share | 1.000 | 1.000 | 0.999 |
|  | (0.001) | (0.001) | (0.001) |
|  |  |  |  |
| 4+ Lenders X Poverty Share | 1.000 | 1.001 | 0.999 |
|  | (0.001) | (0.001) | (0.001) |
|  |  |  |  |
| 1-3 Lenders X Mobility Share | 0.995 | 0.995 | 1.001 |
|  | (0.003) | (0.003) | (0.003) |
|  |  |  |  |
| 4+ Lenders X Mobility Share | 1.006 | 1.007 | 1.004 |
|  | (0.004) | (0.004) | (0.004) |
|  |  |  |  |
| 1-3 Lenders X Urban Share | 0.999 | 0.999 | 1.001** |
|  | (0.000) | (0.000) | (0.000) |
|  |  |  |  |
| 4+ Lenders X Urban Share | 0.998*** | 0.998*** | 1.001 |
|  | (0.001) | (0.001) | (0.000) |
|  |  |  |  |
| Log Population | 3.106*** | 3.212*** | -- |
|  | (0.047) | (0.047) |  |
| Spatial Weights |  |  |  |
| IV: Payday Lender Categories | 1.019 | -- | -- |
|  | (0.015) |  |  |
|  |  |  |  |
| DV: Premature Death Count | 1.050*** | -- | -- |
|  | (0.007) |  |  |
| Spatial Error | -- | 1.031*** | -- |
|  |  | (0.009) |  |
|  |  |  |  |
| State Fixed Effects | Y |  | Y |
| Year Fixed Effects | Y |  | Y |
| N | 56,412 county-years; 3,134 counties | | |

*Notes:* Robust standard errors in parentheses; * p<0.05; ** p< 0.01, *** p<0.001. The spatial OLS models use the log of premature deaths as the dependent variables and includes the log of population ages 20-59 as a control (whereas the population age 20-59 is set as the exposure term in the Poisson regression and not included in controls). When the OLS results are exponentiated, as shown above, they approximate incidence risk ratios. Coefficients from the Poisson model are also reported as incidence risk ratios.

**Appendix B.** Full Results from Random Effects Poisson Regressions Predicting Incidence Risk Ratios for Premature Deaths

|  | M1 | M2 | M3 |
| --- | --- | --- | --- |
| Payday Lender Presence (Ref = 0 Lenders) |  |  |  |
| 1-3 Lenders | 1.009 | 1.011 | 0.616* |
|  | (0.007) | (0.006) | (0.127) |
|  |  |  |  |
| 4 or more lenders | 1.020* | 1.021* | 0.468** |
|  | (0.010) | (0.008) | (0.128) |
| Covariates |  |  |  |
| Share of Male Residents |  | 1.013* | 1.008 |
|  |  | (0.007) | (0.007) |
|  |  |  |  |
| Share of Black/Latinx Residents |  | 1.004* | 1.000 |
|  |  | (0.002) | (0.004) |
|  |  |  |  |
| Share of Residents Below Poverty Line |  | 0.998 | 0.998 |
|  |  | (0.002) | (0.002) |
|  |  |  |  |
| Residential Mobility Share |  | 0.975*** | 0.973*** |
|  |  | (0.003) | (0.004) |
|  |  |  |  |
| Share Urban Residents |  | 0.996*** | 0.996*** |
|  |  | (0.000) | (0.000) |
|  |  |  |  |
| PL X Covariate Interactions |  |  |  |
| 1-3 Lenders X Male Share |  |  | 1.006 |
|  |  |  | (0.004) |
|  |  |  |  |
| 4+ Lenders X Male Share |  |  | 1.013** |
|  |  |  | (0.005) |
|  |  |  |  |
| 1-3 Lenders X Black/Latinx Share |  |  | 0.999 |
|  |  |  | (0.001) |
|  |  |  |  |
| 4+ Lenders X Black/Latinx Share |  |  | 0.999 |
|  |  |  | (0.001) |
|  |  |  |  |
| 1-3 Lenders X Poverty Share |  |  | 1.010*** |
|  |  |  | (0.003) |
|  |  |  |  |
| 4+ Lenders X Poverty Share |  |  | 1.005 |
|  |  |  | (0.004) |
|  |  |  |  |
| 1-3 Lenders X Mobility Share |  |  | 1.001 |
|  |  |  | (0.003) |
|  |  |  |  |
| 4+ Lenders X Mobility Share |  |  | 1.003 |
|  |  |  | (0.004) |
|  |  |  |  |
| 1-3 Lenders X Urban Share |  |  | 1.001*** |
|  |  |  | (0.000) |
|  |  |  |  |
| 4+ Lenders X Urban Share |  |  | 1.001** |
|  |  |  | (0.000) |
|  |  |  |  |
| State Fixed Effects | Y | Y | Y |
| Year Fixed Effects | Y | Y | Y |
| N | 42,230 county-years; 2,626 counties | | |
|  |  |  |  |
| Notes: Exponentiated coefficients; Standard errors in parentheses. * = p < 0.05; ** = p < 0.01; *** = p < 0.001 | | | |

**Appendix C.** Full Results from Random Effects Poisson Regressions, with Regulatory Interactions

|  | M1 | M2 |
| --- | --- | --- |
| Payday Lender Presence (Ref = 0 Lenders) |  |  |
| 1-3 Lenders | 1.010 | 1.003 |
|  | (0.009) | (0.007) |
|  |  |  |
| 4 or more lenders | 1.078*** | 1.056*** |
|  | (0.016) | (0.012) |
|  |  |  |
| Regulatory Strength (Ref = No Regulations) |  |  |
| Weak Regulations | 1.111*** | 1.086*** |
|  | (0.012) | (0.012) |
|  |  |  |
| Moderate Regulations | 1.160*** | 1.104*** |
|  | (0.024) | (0.024) |
|  |  |  |
| Strong Regulations | 1.071*** | 1.047*** |
|  | (0.016) | (0.014) |
| PL X Reg Interactions |  |  |
| 1-3 Lenders X Weak | 0.996 | 1.003 |
|  | (0.009) | (0.009) |
|  |  |  |
| 1-3 Lenders X Moderate | 0.975 | 1.000 |
|  | (0.015) | (0.016) |
|  |  |  |
| 1-3 Lenders X Strong | 0.987 | 1.002 |
|  | (0.016) | (0.015) |
|  |  |  |
| 4+ Lenders X Weak | 0.924*** | 0.947*** |
|  | (0.017) | (0.015) |
|  |  |  |
| 4+ Lenders X Moderate | 0.868*** | 0.919*** |
|  | (0.017) | (0.018) |
|  |  |  |
| 4+ Lenders X Strong | 0.937** | 0.953* |
|  | (0.023) | (0.021) |
|  |  |  |
| *Covariates* |  |  |
| Male Share |  | 1.012 |
|  |  | (0.007) |
|  |  |  |
| Black/Latinx Share |  | 1.004 |
|  |  | (0.002) |
|  |  |  |
| Poverty Share |  | 0.998 |
|  |  | (0.002) |
|  |  |  |
| Mobility Share |  | 0.976*** |
|  |  | (0.003) |
|  |  |  |
| Urban Share |  | 0.996*** |
|  |  | (0.000) |
| N | 42,230 county-years; 2,626 counties | |

Notes: Exponentiated coefficients; Standard errors in parentheses. * = p < 0.05; ** = p < 0.01; *** = p < 0.001
